# Ultrasound vs. Computed Tomography for Severity of Hydronephrosis and Its Importance in Renal Colic

**DOI:** 10.5811/westjem.2017.04.33119

**Published:** 2017-05-15

**Authors:** Megan M. Leo, Breanne K. Langlois, Joseph R. Pare, Patricia Mitchell, Judith Linden, Kerrie P. Nelson, Cristopher Amanti, Kristin A. Carmody

**Affiliations:** * Boston University School of Medicine, Department of Emergency Medicine, Boston, Massachusetts; † Boston Medical Center, Department of Emergency Medicine, Boston, Massachusetts; ‡ Tufts University, Friedman School of Nutrition Science and Policy, Boston, Massachusetts; § Boston University, School of Public Health, Boston, Massachusetts; ¶ New York University School of Medicine, Department of Emergency Medicine, New York, New York

## Abstract

**Introduction:**

Supporting an “ultrasound-first” approach to evaluating renal colic in the emergency department (ED) remains important for improving patient care and decreasing healthcare costs. Our primary objective was to compare emergency physician (EP) ultrasound to computed tomography (CT) detection of hydronephrosis severity in patients with suspected renal colic. We calculated test characteristics of hydronephrosis on EP-performed ultrasound for detecting ureteral stones or ureteral stone size >5mm. We then analyzed the association of hydronephrosis on EP-performed ultrasound, stone size >5mm, and proximal stone location with 30-day events.

**Methods:**

This was a prospective observational study of ED patients with suspected renal colic undergoing CT. Subjects had an EP-performed ultrasound evaluating for the severity of hydronephrosis. A chart review and follow-up phone call was performed.

**Results:**

We enrolled 302 subjects who had an EP-performed ultrasound. CT and EP ultrasound results were comparable in detecting severity of hydronephrosis (*x*^2^=51.7, p<0.001). Hydronephrosis on EP-performed ultrasound was predictive of a ureteral stone on CT (PPV 88%; LR+ 2.91), but lack of hydronephrosis did not rule it out (NPV 65%). Lack of hydronephrosis on EP-performed ultrasound makes larger stone size >5mm less likely (NPV 89%; LR− 0.39). Larger stone size > 5mm was associated with 30-day events (OR 2.30, p=0.03).

**Conclusion:**

Using an ultrasound-first approach to detect hydronephrosis may help physicians identify patients with renal colic. The lack of hydronephrosis on ultrasound makes the presence of a larger ureteral stone less likely. Stone size >5mm may be a useful predictor of 30-day events.

## INTRODUCTION

Renal colic is a common emergency department (ED) presentation and places a significant burden on the healthcare system, with an estimated prevalence affecting 1 in 11 people.^1^ Computed tomography (CT) is considered the imaging gold standard for the diagnosis of renal colic.^2^–^4^ CT has sensitivities of 91–97% and specificities of 91–100% for detecting ureteral stones and also provides information on stone size and location, which can be helpful for predicting successful medical expulsion therapy versus the need for urologic intervention.^3^,^5^–^9^ There are multiple reasons to choose CT imaging selectively in this patient population, most notably to rule out other serious disease such as aortic dissection and other surgical emergencies. However, as many as 50% of patients diagnosed with renal colic will have recurrent episodes and may receive multiple CTs throughout their lifetime, adding to costs, increased length of stay, and radiation exposure.^10^–^13^ There are currently no validated practice guidelines for the diagnosis and ED management of renal colic; thus, the need for a multidisciplinary approach to managing this disease is clear.^12^,^14^–^17^. The role of emergency physician- (EP) performed ultrasound (US) in the management of patients with renal colic has recently gained more attention, but its incorporation into an accepted algorithm remains debatable^18^–^21^

**Figures 1a, 1b, 1c:**
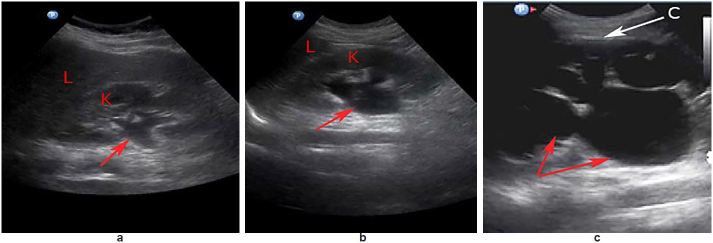
Hydronephrosis visualized as an anechoic black area on ultrasound. Figure 1a: mild hydronephrosis (red arrow) showing dilation of the proximal renal pelvis of the kidney (K), liver (L); Figure 1b: moderate hydronephrosis (red arrow) showing dilation of the renal pelvis and calyces of the kidney (K), liver (L); Figure 1c: severe hydronephrosis showing large dilation of the renal pelvis and calyces (red arrows) extending outward and resulting in a thinning of the renal cortex (C).

US has the advantage of using no radiation, and research continues to support its role in the diagnosis and management of renal colic in the ED.^22^ The low sensitivity of US for identifying stone size and stone location may limit its usefulness in predicting the clinical course or follow-up planning for patients with renal colic.^23^ However, hydronephrosis is easily detected by US and its presence or absence may provide physicians with useful information to assist in renal colic management. US has been shown to have sensitivities ranging from 72–87% and specificities between 73–83% in the detection of hydronephrosis when compared to CT.^24^–^26^ Hydronephrosis is a secondary sign of ureteral calculi and is a dilation of the renal pelvis and calyces (Figures 1a, 1b, 1c).

Hydronephrosis can be identified by EPs with various levels of US experience, with a moderate degree of hydronephrosis yielding a higher specificity.^27^ The clinical significance of hydronephrosis is still unclear, although some have suggested that hydronephrosis may be a predictor of stone size and the need for urologic intervention or hospitalization.^17^,^28^–^32^ If EPs are to implement an “ultrasound-first” approach, it is important to know the test characteristics of hydronephrosis detected by EP-performed US for the diagnosis of renal colic and whether there is any predictive value for 30-day events.

The primary goal of this study was to determine if EP-performed US can detect severity (none, mild, moderate, severe) of hydronephrosis in ED patients with suspected renal colic when compared to CT. We also sought to determine the diagnostic test characteristics of hydronephrosis detected by EP-performed US for the presence of a ureteral stone and ureteral stone size > 5mm. A secondary goal of this study was to generate hypotheses regarding predictors of 30-day events in renal colic patients.

## MATERIAL AND METHODS

This was a prospective, observational cohort study of a convenience sample of ED patients with suspected renal colic from November 2010 to March 2014. The study was performed at an urban academic medical center with over 130,000 annual visits. The Boston Medical Center and Boston University Medical Campus Institutional Review Board approved this study. Eligible patients were identified for inclusion by either a trained research assistant (RA) or a physician investigator. RAs were available to screen the department electronic medical record system for potential eligible patients Monday-Friday from 8:00 am–11:00 pm. Enrollment occurred during periods when an EP investigator was able to perform the US. Patients were approached if they met inclusion criteria: age ≥21; CT of the abdomen and pelvis without contrast ordered; and ability to provide a telephone number for 30-day follow-up. We excluded prisoners, non-English speaking patients and those unable to provide informed consent (defined as medically unstable, those who had dementia, altered mental status, or deemed mentally incompetent by the treating physician). Written informed consent was obtained from all study participants. Participants were excluded if an US could not be completed prior to discharge from the ED.

**Population Health Research Capsule**

What do we already know about this issue?

Renal colic will affect 1 in 11 people and 50% have a recurrence. CT scan is the imaging choice for urologic management of renal colic but multiple CT scans can be costly and have health risks.

What was the research question?

Can EP ultrasound detect the degree of hydronephrosis as compared to CT scan? Does hydronephrosis diagnose a ureteral stone or predict renal colic outcomes?

What was the major finding of the study?

EP Ultrasound can diagnose the degree of hydronephrosis as compared to CT. Larger ureteral stones are less likely present when there is no hydronephrosis. Larger ureteral stone may predict important renal colic outcomes.

How does this improve population health?

Using an EP ultrasound first approach is reasonable and may help avoid the need for a CT scan in renal colic patients on the day of their ED visit.

After consent was obtained, one of the investigators, blinded to CT results, performed an US and completed a standardized data collection sheet. All video images were obtained with a Philips (Amsterdam, Netherlands) HD11 XE machine, a Philips Sparq machine, or a Zonare (Mountain View, CA, USA) z.one *ultra sp* machine using a curvilinear probe (6–2 MHz). Fourteen EP investigators participated in this study and performed all of the ultrasounds. The principal investigator (PI) of the study was the emergency ultrasound director, who had formal fellowship training. Three of the investigators were attendings and 11 were residents throughout the study enrollment period. The physician investigators were required to complete minimum training requirements before participating in this study, which included satisfying the 2008 American College of Emergency Physician (ACEP) Ultrasound Guidelines.^33^. In addition, the PI of the study trained all investigators how to obtain proper renal US views and how to classify the severity of hydronephrosis to ensure uniformity. All US images were recorded and later reviewed by the PI, who was blinded to clinical information and outcome data. The review ensured adequate image acquisition and interpretation and was used to assess inter-rater reliability.

### Study Protocol

The study protocol required both long- and short-axis views of each kidney. The US findings recorded on the data sheet were hydronephrosis (none, mild, moderate, severe). Color Doppler was used to differentiate mild hydronephrosis from the confluence of vessels in the renal pelvis. The CT parameters recorded were hydronephrosis and/or hydroureter (none, mild, moderate, severe); renal stone location and size; and any additional pathological findings. The PI reviewed the final reading on all CT imaging to ensure accuracy. The reading was considered final when a dictated report by an attending radiologist appeared in the medical record.

In our study, we defined renal colic as one of the following: 1) CT-confirmed ureteral stone; 2) the presence of CT findings confirming the recent passage of a stone as dictated on the radiology report, which included hydronephrosis, hydroureter, perinephric or periureteric stranding or stone in the bladder; 3) attending clinical impression or discharge diagnosis of renal colic obtained from review of the medical chart. The medical records were reviewed for disposition, hospital discharge diagnosis and return events. In cases where the medical record showed no return outcome within 30 days, an investigator or RA conducted a structured follow-up phone call to gather information directly from the participant. Two EP investigators, who were attending physicians, reviewed all medical records to ensure accurate data extraction and to confirm the diagnosis of renal colic and 30-day events. The simple kappa of agreement was reported. If there were any discrepancies between the two reviewers, these were resolved by the review of a third EP attending physician investigator.

### Primary Data Analysis

We needed 273 participants in order to evaluate the primary goal of comparing EP-performed US with CT in identifying the severity of hydronephrosis (none, mild, moderate, severe) with a chi-square test. This sample size calculation was made using a chi-square test of independence with three degrees of freedom, looking for a minimum effect size of 0.2 with 80% power to detect whether the EP US findings yielded the correct classifications as determined by the corresponding gold standard CT. CT interpreted by an attending radiologist blinded to EP US findings was the criterion standard for diagnosing presence or absence of hydronephrosis and its severity. We also performed a Wilcoxon signed-rank test to further assess whether differences existed between the EP-performed US and CT classifications of hydronephrosis. An ROC (receiver operator curve) was drawn and the area under the curve calculated to assess the ability of US to correctly classify those patients with and without hydronephrosis, using CT as the criterion standard. We calculated the diagnostic test characteristics (sensitivity, specificity, positive predictive value [LR+), and negative predictive value, [LR−]) of any degree of hydronephrosis on EP-performed US for the presence of any ureteral stone or ureteral stone size > 5mm on CT.

For the secondary objective of assessing predictors of 30-day events in participants with confirmed renal colic, a binary outcome measure was used and defined as the following: admitted to the hospital on the day of enrollment due to renal colic; or return visit for pain, infection, the need for a urologic procedure, or hospital admission related to renal colic. We analyzed four different models to generate hypotheses on the association of 30-day events among renal colic patients with EP-performed US or CT findings. Four simple logistic regression models were fit using the following as independent variables: Model 1) any hydronephrosis on EP US; Model 2) severity of hydronephrosis on EP US categorized as none, mild, moderate or severe; Model 3) ureteral stone, size ≥ 5mm on CT; and Model 4) proximal ureteral stone location on CT. The 30-day event outcomes used for each of the four models were defined as admission at initial ED visit or return visit within 30 days for pain, infection, GU procedure, or hospital admission (related to pain, infection, or planned urologic procedure). We calculated odds ratios (OR) and 95% confidence intervals (CI) for each model. We used 95% CIs and *P* values to determine significance at the 0.05 level. All analyses were done in SAS (version 9.3; SAS Institute, Inc., Cary, NC).

## RESULTS

**Figure 2:**
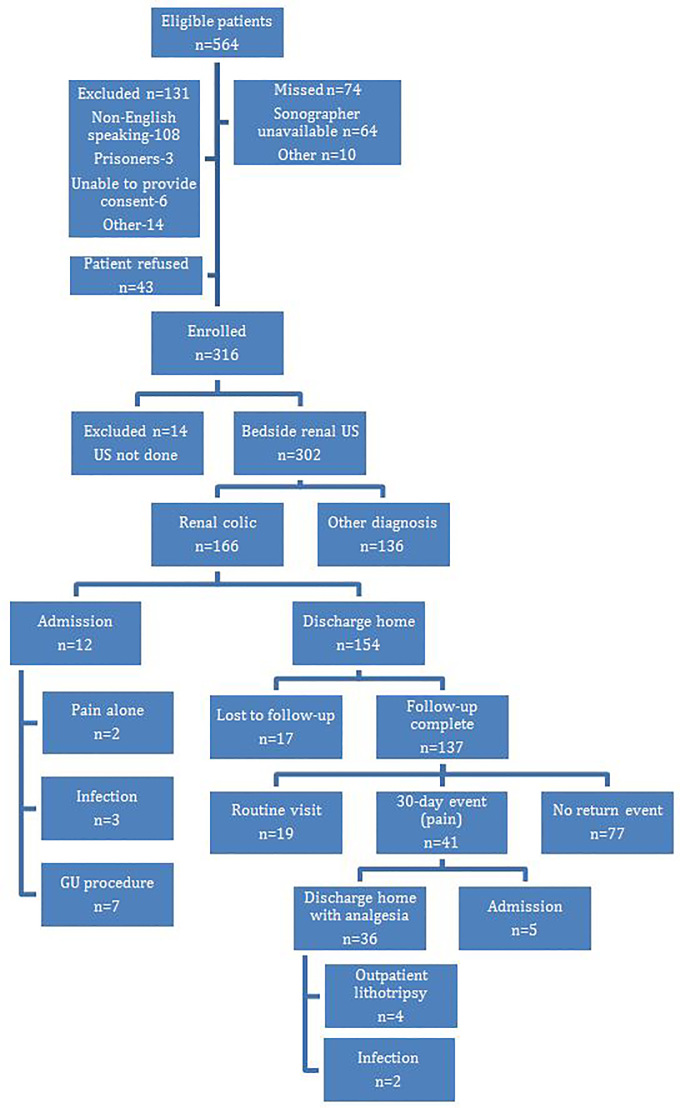
Flowchart of enrollment figures in study comparing ultrasound and computed tomography for detection of severity of hydronephrosis. *GU,* genitourinary.

Between November 2010 and March 2014, 564 eligible participants with suspected renal colic were evaluated in the ED and 316 were enrolled (Figure 2). We excluded an additional 14 participants due to the US not being performed prior to the patient leaving the ED, leaving 302 participants for analysis (Table 1).

Our results show that EP-performed US can detect the severity of hydronephrosis when compared to CT as the gold standard, (chi-square p<0.001) (Table 2). Of the 302 participants, five were missing CT results for the classification of hydronephrosis severity, which left 297 included in the analysis. In comparing EP-performed US to the criterion standard CT in the detection of the severity of hydronephrosis, the area under the curve using ROC analysis was 88.3%. A Wilcoxon signed-rank test determined that there was a statistically significant median difference between the CTs and EP-performed US classifications of hydronephrosis, *W* = −312.5, *p* =0.03, with ultrasound under-classifying the severity in 9% of participants, over-classifying in 13%, and correctly classifying in 78%. The majority of misclassified degrees of hydronephrosis by US were off by one degree of severity (Table 2).

The PI reviewed all recorded ultrasounds, and the inter-rater agreement between the PI interpretation of hydronephrosis and all other investigators was 91% with a weighted kappa of 0.86. The test characteristics for detection of hydronephrosis are displayed in Table 3a,b. The detection of any hydronephrosis on EP-performed US had a sensitivity of 85%, a specificity of 71%, a LR+ = 2.91, and a LR− = 0.22 for the presence of any ureteral stone visualized on CT. For the presence of a ureteral stone >5mm on CT, the detection of any hydronephrosis by EP- performed US had a sensitivity of 86%, a specificity of 37%, a LR+ = 1.36, and a LR− = 0.39.

Of the 302 participants who had an EP-performed US, 166 (55%) had a diagnosis of renal colic based on our study definition and 136 had an alternate diagnosis by CT (Figure 2). There was 96% agreement between the two physician reviews of the 302 charts, with 13 discordant charts that required a tiebreaker review by a third physician investigator (simple kappa=0.91[0.87, 0.96]). Of the 166 participants with a diagnosis of renal colic, 128 had a stone visualized on CT, 15 had no stone visualized but had signs of a recently passed stone on CT, and 23 had an ED attending clinical impression or discharge diagnosis of renal colic on chart review. There were 39 (13%) participants who had some other diagnostic findings on CT, including 21 (7%) who required additional management. Significant pathology included diverticulitis (5), malignancy-related findings (7), non-specific mesenteric inflammatory findings (5), chronic pancreatitis (1), small bowel obstruction (1), pneumonia (1), and common bile duct and pancreatic duct dilation (1). The remaining 97 patients who did not have renal colic had no other pathology identified on CT.

Of the 166 participants who had renal colic, 12 were admitted to the hospital. One admitted participant went to the intensive care unit for urosepsis and had severe hydronephrosis on CT that was correctly identified on EP US. The remaining 154 participants with renal colic were discharged home from the ED and had a 30-day medical record review and/or phone call performed. Information abstracted during chart review included the presence or absence of one of the following return events within 30 days: a return visit for continued pain; infection; the need for urologic intervention; or hospital admission related to renal colic. Seventeen participants were lost to 30-day follow-up due to lack of information in the chart review and inability to contact by phone call. Of the remaining 137 participants included in the follow-up cohort, 77 had no further events, 19 had a routine visit and 41 had a 30-day return event (Figure 2).

The cohort used in the hypothesis-generating secondary analysis of predictors of 30-day events related to renal colic were the 12 patients admitted to the hospital on the day of the ED visit and the 137 participates who were discharged home with a diagnosis of renal colic and had a completed 30-day follow up (n=149). We performed four logistic regression models to analyze factors that may be predictive of 30-day events. These factors included the presence of hydronephrosis on EP-performed US, the degree of hydronephrosis on EP-performed US, the presence of a ureteral stone > 5mm, and proximal ureteral stone location on CT (Table 4). Of these four exploratory models, we found a significant association only for the presence of a ureteral stone > 5mm. Renal stones > 5mm had an OR of 2.30 for a 30-day event compared to smaller stones, 20 out of 40 vs. 33 out of 109 (95% CI [1.10, 4.84]; p=0.03).

## DISCUSSION

This study supports the findings of prior research that EP-performed US can reliably identify the severity of hydronephrosis when compared to CT as the criterion standard 24–30. We found that any degree of hydronephrosis on EP US makes the presence of a ureteral stone on CT more likely (PPV 88%, LR+ 2.91), but a lack of hydronephrosis did not rule out the diagnosis (negative predictive value [NPV] 65%, LR− 0.22). Prior studies show that 4–8% of patients with renal colic will not have any secondary signs of ureteral obstruction, such as hydronephrosis, on imaging.^34^,^35^ The diagnosis should still be considered in cases with high enough clinical suspicion, and CT can be performed to confirm the diagnosis if deemed necessary by the treating physician. Many EPs caring for patients with renal colic will order a CT to rule out other significant diagnoses and report feeling more confident when a CT is performed.^36^ This study found an incidence of other findings on CT to be 7%, which is consistent with other research that has reported CT-diagnosed incidental findings in 3–12% of patients imaged for renal colic.^37^–^39^ When physicians are deciding on imaging in the ED, acceptable risk tolerance for missing an important alternate diagnosis still needs to be considered.

Prior studies have suggested that hydronephrosis is more sensitive and specific for identifying larger stones.^28^–^30^ We did find that hydronephrosis on EP-performed US had a high sensitivity (85.7%) for a stone > 5mm on CT, but it did not have a high specificity (37.1%) or LR+ (1.36) to rule in larger stones. However, we found that the absence of hydronephrosis on EP US is good for ruling out the presence of stones > 5mm (NPV 88.5%, LR− 0.39) and may reassure the provider that a large stone is not present.

What remains unclear is whether or not the severity of hydronephrosis provides additional predictive information in patients with renal colic. Most patients who present to the ED with renal colic have few adverse events within a follow-up period of 180 days.^16^–^40^ We chose to perform follow up at 30 days based on the recommended trial of medical expulsion therapy of 4–6 weeks.^8^,^10^,^41^,^42^ Two prior studies found moderate and severe hydronephrosis to be more predictive of the need for urologic intervention.^17^,^32^ A smaller prospective study analyzed the test characteristics of severity of hydronephrosis and stone size >5mm on risk of 30-day hospitalization in renal colic patients and found any hydronephrosis to be 100% sensitive and 44% specific.^31^ For our renal colic outcomes measure, we defined 30-day events as admission to the hospital on the day of enrollment or 30-day return for admission related to renal colic, urologic intervention, pain control, or infection based on prior definitions of adverse outcomes in the literature and clinical factors we felt most important to EPs evaluating these patients in the ED^16^,^17^,^31^,^32^. In addition to examining the predictive value of hydronephrosis on EP US for return events, we looked at the predictive value of proximal ureteral stone location and ureteral stones ≥5mm identified on CT due to research suggesting these factors lead to an increased likelihood of requiring urologic intervention.^7^,^9^,^43^ In this study, the presence of any degree of hydronephrosis on EP US was not predictive of a 30-day event. In addition, the presence of moderate or severe hydronephrosis was also not predictive of 30-day events. There may be other factors contributing to the severity of hydronephrosis, such as the length of time the stone has been present or a patient’s hydration status. If a larger stone size is not associated with greater degrees of hydronephrosis, then the severity of hydronephrosis may not be predictive of 30-day events, as prior studies have suggested. This also implies that misclassifying the degree of hydronephrosis on US compared to CT may not be clinically relevant. Therefore, it may be reasonable to ask an EP to identify the presence or absence of hydronephrosis alone without attempting to differentiate the degree.

This study did find that larger stone size > 5mm on CT was a statistically significant (P= 0.03) predictor of a 30-day event. This is consistent with prior studies that show that larger stones are less likely to pass without intervention.^7^,^41^,^43^ A more proximal stone location had 2.08 times the odds of a 30-day event, but it was not statistically significance. The secondary analysis in this study is underpowered and limited by small sample size; therefore, larger multi-center studies are needed for further investigation.

From an observational perspective, this study found that all subjects (41) with a 30-day event had one due to continued pain. Of these subjects, only five were admitted and later discharged without further complications. If most return events are due to continued pain, which is the natural course of this disease, we need to question the utility of obtaining a CT on every patient who presents to the ED with the suspected diagnosis. US is a reasonable first-line screening modality in suspected renal colic patients, especially if further research confirms the predictive value of hydronephrosis in detecting any or larger ureteral stones. Using an “ultrasound-first” approach can help decrease potential radiation exposure, costs and prolonged lengths of stay in the ED.

## LIMITATIONS

This was a single-site study performed at an academic ED with an emergency medicine residency and an active US section. These results may not be generalizable to other clinical settings and may not be easily reproducible at institutions where EPs lack equivalent training in the use of US. This study was a convenience sample based on availability of the EP investigators and RAs. Inclusion criteria required participants to receive a CT, which may have introduced a selection bias in the population studied by missing potential subjects with suspected renal colic who had no imaging. Also, although the literature supports CT as the gold standard for evaluation of renal colic it is possible that CT may be an imperfect gold standard for identifying the severity of hydronephrosis. This may introduce an imperfect gold standard bias when evaluating the test characteristics of EP-performed US in classifying severity of hydronephrosis.

The physician investigators were residents and attendings who may have had varying degrees of training in performing renal US; however, all investigators had met ACEP minimum standards and had uniform training in the renal US protocol.

We included attending clinical impression or discharge diagnosis of renal colic on chart review in the definition of renal colic because this disease is often a clinical diagnosis and negative imaging may have been a result of a recently passed stone. The intention was to not miss the group of patients who would be managed by physicians as renal colic despite negative imaging. This may have introduced information bias in our study. Efforts were made to ensure accuracy of data extraction from the chart review by having two separate physician investigators blindly review all medical information pertaining to the ED visits. The reviewers, however, were not blinded to the hypothesis of the study. The follow-up period of 30 days may also be a limitation to our results, with longer periods of follow-up revealing more events. The interpretation of our analysis of a return event is limited by sample size and not powered for this study. This analysis was intended to be hypothesis generating. Further research with a larger sample size is required to determine significant predictors of 30-day events in patients with renal colic.

## CONCLUSION

EP-performed ultrasound can identify the severity of hydronephrosis in patients with suspected renal colic compared to CT. The diagnostic test characteristics of hydronephrosis detected by EP-performed US indicates that any degree of hydronephrosis is a good predictor for the presence of a ureteral stone on CT, but may be less reliable in identifying larger stones than previously reported in the literature. The lack of hydronephrosis on EP-performed US should not be used to rule out the presence of a ureteral stone but it does makes the presence of a larger stone less likely. This may be helpful in risk stratifying patients for a return event and prioritize appropriate follow-up. Our follow-up revealed that most patients who have a 30-day return event for renal colic come back for continued pain rather than more serious morbidity, and therefore CT may not be needed on all patients presenting to the ED with suspected renal colic. Although EP-performed ultrasound is a reasonable first-line screening tool in suspected renal colic, CT may still be warranted in high-risk patients or in those with suspicion for an alternate diagnosis. Larger multi-centered studies are needed to further explore these predictors in renal colic patients.

## Supplementary Information





## Figures and Tables

**Table 1:** Descriptive summary of participants, n=302, in a study comparing ultrasound and computed tomography for detection of degree of hydronephrosis.

Demographic characteristics	n (%)
Age, years (mean ± SD (median))	43.1 ± 13.6 (43)
Gender	
Male	170 (56.3)
Female	132 (43.7)
Race	
White/non-Hispanic	90 (29.8)
Black/African American	111 (36.8)
Hispanic	76 (25.2)
Asian	6 (2.0)
Other	19 (6.3)

**Table 2:** Comparison of emergency-physician-performed ultrasound and computed tomography in the detection of degree of hydronephrosis, n=302.

ED ultrasound classification	CT scan classification (frequency missing=5)					Correct	Incorrect	Total

	None	Mild	Moderate	Severe	Total			
None	160	14	0	0	174	160	14	174
Mild	18	46	11	0	75	46	29	75
Moderate	6	16	24	1	47	24	23	47
Severe	0	0	0	1	1	1	0	1
Total	184	76	35	2	297	231	66	297

*ED*, emergency department; *CT*, computed tomography.

Chi-squared = 51.7; df = 3; p-value = <0.001.

**Table:** 

a.		

	CT positive for any ureteral stone	CT negative for ureteral stone
Hydronephrosis detected by EP ultrasound (mild/moderate/severe)	100	14
No hydronephrosis detected by EP ultrasound	18	34

*CT*, computed tomography; *CI*, confidence interval; *LR*, likelihood ratio.

Sensitivity=84.8% (95% CI [78.3, 91.2]); Specificity=70.8% (95% CI 58, 83.7);

Positive Predictive Value=87.8% (95% CI [81.7, 93.7]); Negative Predictive Value=65.4% (95% CI [52.5, 78.3]); LR+=2.91 (95% CI [1.6, 4.21]); LR−=0.22 (95% CI [0.12, 0.32]).

**Table:** 

b.		

	CT positive for any ureteral stone >5mm	CT negative for ureteral stone >5mm
Hydronephrosis detected by EP ultrasound (mild/moderate/severe)	100	14
No hydronephrosis detected by EP ultrasound	18	34

*CT*, computed tomography; *CI*, confidence interval; *LR*, likelihood ratio.

Sensitivity=85.7% (95% CI [71.5, 94.6]); Specificity=37.1% (95% CI [28.6, 46.2]);

Positive Predictive Value=31.6% (95% CI [23.2, 40.95]); Negative Predictive Value=88.5% (95% CI [76.6, 95.65]); LR+=1.36 (95% CI [1.13, 1.64]); LR−=0.39 (95% CI [0.18, 0.84]).

**Table 4:** Exploratory analysis of predictors of 30-day events, n=149†‡.

	OR (95% CI)	p-value
Model 1		
Any hydronephrosis on ED ultrasound (no hydronephrosis is ref.)	1.39 (0.66, 2.93)	0.38
Model 2		
Severity of hydronephrosis on ED ultrasound (no hydronephrosis is ref.)		
Mild	1.28 (0.57, 2.88)	0.55
Moderate or severe	1.59 (0.65, 3.89)	0.31
Model 3		
Obstructing stone, size ≥ 5mm^§^ (no is ref.)	2.30 (1.10, 4.84)	0.03
Model 4		
Proximal stone location (no is ref.)	2.08 (0.88, 4.89)	0.09

*ED*, emergency department.

† Outcome defined as admission at initial emergency department (ED) visit or return to ED or clinic within 30 days for pain, infection, genitourinary (GU) procedure, or hospital admission (related to pain, infection, or planned GU procedure); probability modeled is outcome = ‘yes’.

‡ Those who did not have renal colic or were lost to follow-up were excluded from this analysis.

§§ Obstructing stone, determined by CT scan, defined.
