# Supplementary material for: Ultrasound vs. Computed Tomography for Severity of Hydronephrosis and Its Importance in Renal Colic
Source: West J Emerg Med. 2017 May 15;18(4):559–68. doi: 10.5811/westjem.2017.04.33119 (PMC5468059; doi:10.5811/westjem.2017.04.33119)
Supplement: Supplementary file 1 [file wjem-18-559-s001.docx]

**Appendix 1. Wilcoxon Signed Rank Test comparing ordinal rankings of hydronephrosis between paired samples of U/S and CT, n=302**

|  | **CT Ranking^*^** | **ED Ranking^*^** | **Difference**  **(CT Ranking – ED Ranking)** |
| --- | --- | --- | --- |
| *Median (min, 25%, 75%, max)* | 0 (0, 0, 1, 3) | 0 (0, 0, 1, 3) | 0 (-2, 0, 0, 1) |
| *Freq. Missing* | 5 | 0 | 5 |
|  |  |  |  |
| **Signed Rank Statistic = -312.5; p-value = 0.03** | | | |

^*^Ordinal Scale: 0=None, 1=Mild, 2=Moderate, 3=Severe
