# Supplementary material for: Ultrasound vs. Computed Tomography for Severity of Hydronephrosis and Its Importance in Renal Colic
Source: West J Emerg Med. 2017 May 15;18(4):559–68. doi: 10.5811/westjem.2017.04.33119 (PMC5468059; doi:10.5811/westjem.2017.04.33119)
Supplement: Supplementary file 2 [file wjem-18-559-s002.doc]

**Appendix 2. Table showing U/S test result (none/mild/mod/severe) versus gold standard CT scan (where no disease D- = "none", and disease D+ = "mild,moderate or severe")**

|  | | **Ultrasound Result (Test)** | | | |  |
| --- | --- | --- | --- | --- | --- | --- |
|  |  | **none (1)** | **mild (2)** | **mod (3)** | **severe (4)** | **Total** |
| **CT Scan**  **Gold Standard** | **None (no disease)** | 160 | 14 | 0 | 0 | 174 |
|  | **Mild,moderate, severe (diseased)** | 24 | 62 | 35 | 2 | 123 |
|  | **Total** | 184 | 76 | 35 | 2 | 297 |

ROC curve is as follows. It includes the CT scan results as a binary outcome (diseased/non-diseased).
